# Microparticle and interleukin-1β production with human simulated compressed air diving

**DOI:** 10.1038/s41598-019-49924-1

**Published:** 2019-09-16

**Authors:** Kaighley D. Brett, Nathan Z. Nugent, Noelle K. Fraser, Veena M. Bhopale, Ming Yang, Stephen R. Thom

**Affiliations:** 1 Canadian Forces Health Services Center, Canadian Armed Forces, Ottawa, Canada; 2 Departments of Emergency Medicine, University of California, San Diego, USA; 3 University of Maryland School of Medicine, Baltimore, USA

**Keywords:** Adaptive immunity, Cardiovascular biology

## Abstract

Production of blood-borne microparticles (MPs), 0.1–1 µm diameter vesicles, and interleukin (IL)-1β in response to high pressure is reported in lab animals and associated with pathological changes. It is unknown whether the responses occur in humans, and whether they are due to exposure to high pressure or to the process of decompression. Blood from research subjects exposed in hyperbaric chambers to air pressure equal to 18 meters of sea water (msw) for 60 minutes or 30 msw for 35 minutes were obtained prior to and during compression and 2 hours post-decompression. MPs and intra-particle IL-1β elevations occurred while at pressure in both groups. At 18 msw (n = 15) MPs increased by 1.8-fold, and IL-1β by 7.0-fold (p < 0.05, repeated measures ANOVA on ranks). At 30 msw (n = 16) MPs increased by 2.5-fold, and IL-1β by 4.6-fold (p < 0.05), and elevations persisted after decompression with MPs elevated by 2.0-fold, and IL-1β by 6.0-fold (p < 0.05). Whereas neutrophils incubated in ambient air pressure for up to 3 hours *ex vivo* did not generate MPs, those exposed to air pressure at 180 kPa for 1 hour generated 1.4 ± 0.1 MPs/cell (n = 8, p < 0.05 versus ambient air), and 1.7 ± 0.1 MPs/cell (p < 0.05 versus ambient air) when exposed to 300 kPa for 35 minutes. At both pressures IL-1β concentration tripled (p < 0.05 versus ambient air) during pressure exposure and increased 6-fold (p < 0.05 versus ambient air) over 2 hours post-decompression. Platelets also generated MPs but at a rate about 1/100 that seen with neutrophils. We conclude that production of MPs containing elevated concentrations of IL-1β occur in humans during exposure to high gas pressures, more so than as a response to decompression. While these events may pose adverse health threats, their contribution to decompression sickness development requires further study.

## Introduction

Decompression sickness (DCS) presents a health risk for deep sea divers and a constraint to space exploration and provocative diving operations. Bubbles are thought to play a role in pathophysiological responses mediating DCS, however, they are often produced without symptoms^1–3^. Therefore, additional etiological factors have been sought to explain DCS development. Microparticles (MPs) are membrane vesicles with diameters of 0.1 to 1.0 µm generated by a variety of cells, and they are elevated in association with simulated as well as *bona fide* underwater diving^4–11^. Actions that decrease the incidence of DCS also diminish MPs production^9,10^. Murine studies support a role for MPs in high pressure gas pathophysiology and possibly with gas bubble nucleation^12–15^.

In the mouse DCS model, neutrophil activation and associated systemic inflammatory events are initiated by MPs^11–15^. Vascular damage and prolongation of nerve action potential seen in decompressed animals can be recapitulated by injecting decompression-induced MPs into naïve mice^13–15^. Recently, particular attention has been focused on interleukin (IL)-1β because the synthetic pathways for MPs production overlap with those required for activation of the nucleotide-binding domain, leucine-rich-containing family, pyrin domain-containing-3 (NLRP3) inflammasome that generates IL-1β^16–18^. IL-1β is synthesized without a leader peptide, so cannot utilize the conventional secretory pathway and requires packaging into vesicles for secretion^16,19–21^. Vascular injuries mediated by MPs following some insults can be directly linked to high concentrations of IL-1β in the particles^11,16–18^.

This investigation was prompted because translation of findings from the murine decompression model to humans requires additional study. MPs elevations have been shown in divers, with some sub-types, such as those from neutrophils and platelets, being significantly higher in individuals suffering from DCS than in asymptomatic divers^22^. However, no investigation has been done examining an association between MPs and IL-1β in human divers. Additionally, the time course for increases in MPs production needs further study because in mice it appears to be initiated during the high pressure exposure, rather than a phenomenon that develops after decompression^11^. Recent studies suggest that MPs may provide an explanatory link between bubbles and DCS^4,8,22^. With these issues in mind, we obtained blood from research subjects before, during, and after simulated dives in a hyperbaric chamber. The goal was to assess relationships among MPs, neutrophil responses, and IL-1β when research subjects were pressurized with air to the equivalent of 18 and 30 meters of sea water (msw) as well as to decompression.

## Results

### Research subjects

40 research subjects for high pressure investigations were recruited to the study between October 2016 and November 2018. Data for 9 subjects was removed due to collection errors such as inadequate volume and clotting of samples. Samples for 15 subjects were adequate for analysis related to the 18 msw exposures. Samples from 16 subjects were used in analyses from the 30 msw exposures. MP and neutrophil activation analyses were performed on all samples. IL-1β analysis was not considered until 2018 after the role for the cytokine had been shown in murine studies^11^, so this analysis was only performed on samples from the February 2018 study to 18 msw (n = 6) and the November 2018 study to 30 msw (n = 6). All study subjects were males with a mean age of 35.5 ± 2.4 (SE) years old. An additional 8 subjects, 4 female with a mean age of 40.5 ± 4.4 (SE) were recruited in June 2019 to provide blood samples for *ex vivo* investigations.

### MPs elevations

**Figure 1:**
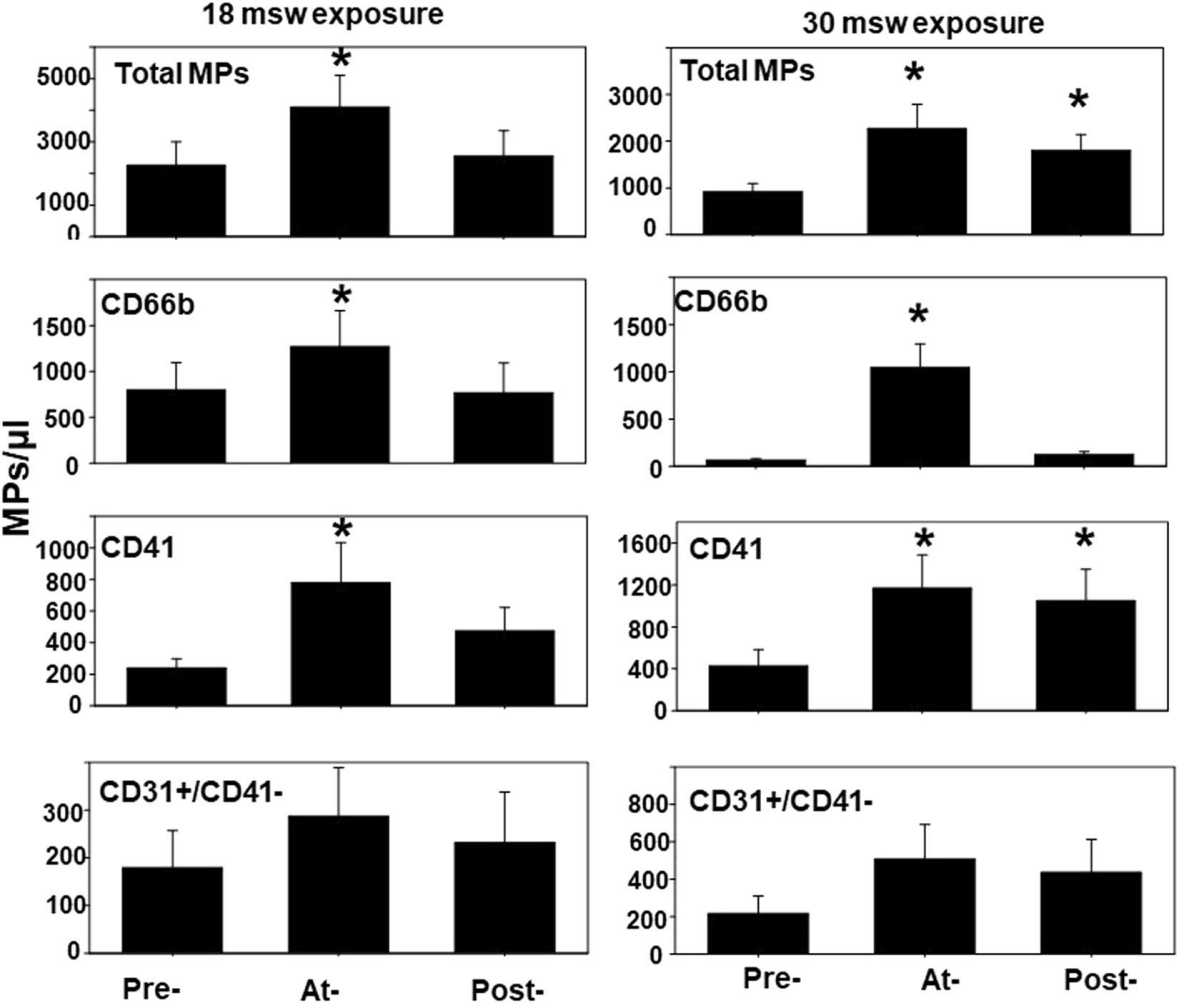
Changes in microparticles. Blood borne MPs were quantified pre-, at- and post-exposures to 18 or 30 msw as described in Methods. Flow cytometric measurements were made to quantify the number of all 0.3 to 1 µm diameter Annexin V-positive particles (top frame), as well as those expressing proteins specific to certain cells: CD66b (mature neutrophils), CD41 (platelets), and CD31+/CD41−dim (endothelium). Data are mean ± SE, n is shown for each sample, * indicates significantly different from pre-exposure, p < 0.05, RM ANOVA.

Results from MPs analysis are shown in Fig. 1. Each subject’s intra-dive and post-decompression data were compared against their pre-dive findings using repeated measures ANOVA on ranks. Statistically significant elevations were present in both dive groups for intra-dive total MPs and sub-groups expressing surface proteins from neutrophils (CD66b) and platelets (CD41), and for total and CD41-expressing MPs post-dive in the 30 msw group. Changes in the number of MPs expressing CD31 but not CD41, and thus thought to be generated by endothelium, were not statistically significantly different across the experiment. This differs from human and animal studies where endothelial injury has been documented^11,17,23–25^.

### Neutrophil activation

**Figure 2:**
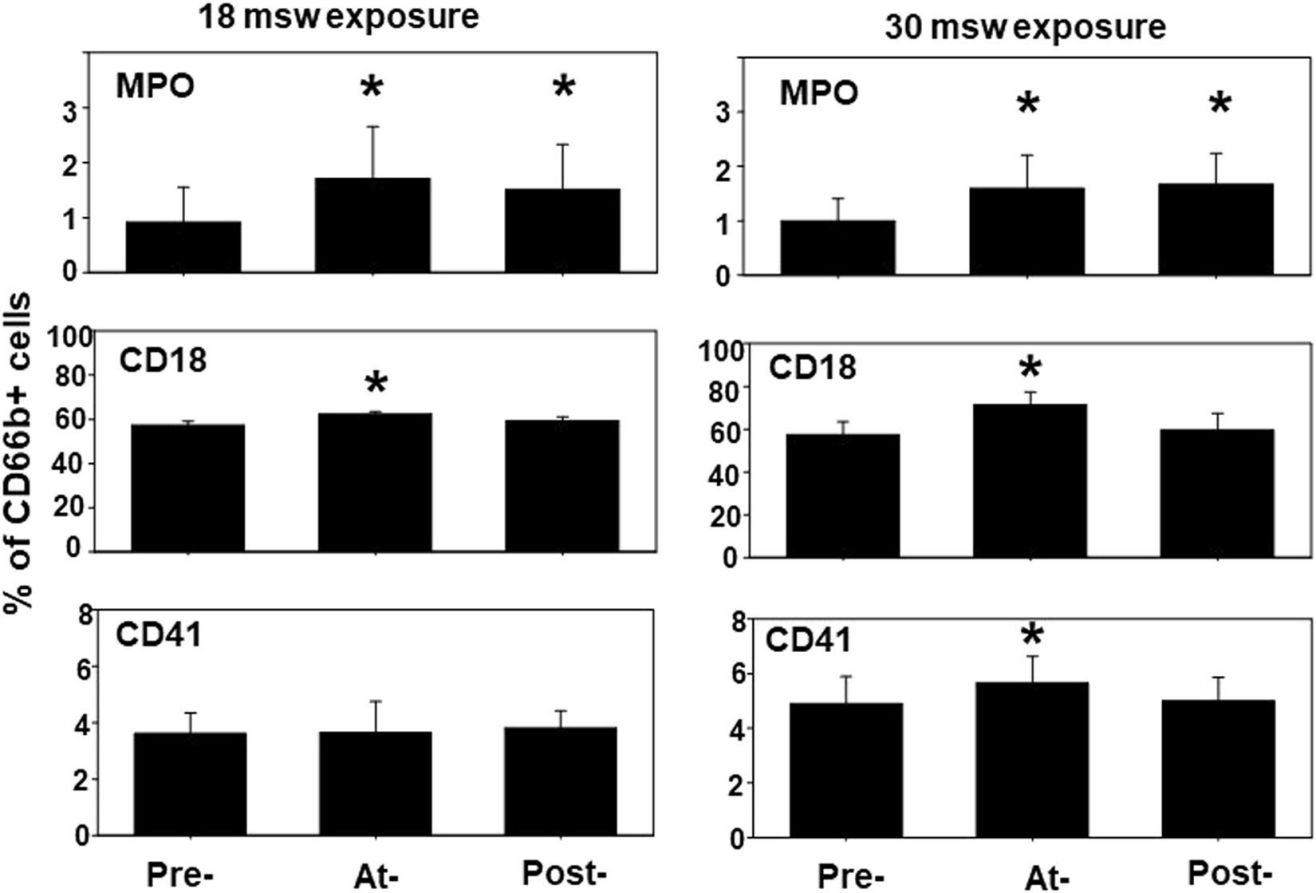
Neutrophil activation. Surface proteins on neutrophils were quantified in blood samples as described in the caption for Fig. 1. Flow cytometry analyses of CD66b-positive cells (neutrophils) assessed surface expression of myeloperoxidase (MPO) and CD18, and platelet-specific CD41 as reflecting platelet-neutrophil interactions. Data are mean ± SE, n is shown for each sample, * indicates significantly different from pre-exposure, p < 0.05, RM ANOVA.

Neutrophil activation, shown in Fig. 2, was assessed as CD66b-positive cell populations expressing myeloperoxidase (MPO) and an elevated amount of CD18, a component of β_2_ integrins. One mechanism for neutrophil activation involves interactions with platelets or platelet-derived particles^12^. To assess whether platelet-neutrophil interactions were present, surface expression of CD41 was measured on the CD66b + cells. Evidence for neutrophil activation was found during and after both the 18 and 30 msw exposures, and evidence of platelet-neutrophil interactions was present during, but not after, the 30 msw exposure.

### MPs and IL-1β content

**Figure 3:**
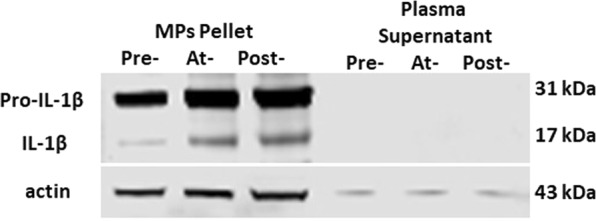
Representative Western blot of MPs and supernatant plasma fraction. Plasma samples from one research subject exposed to 30 msw were prepared as described in Methods to separate 10,000 MPs from suspending plasma. SDS buffer was added to the MPs pellet and supernatant fractions to maintain exact comparisons, and 20 µg protein was loaded to each lane. Data show entire, uncropped blots that had been cut so that individual antibodies could be used for probing pro- and mature forms of IL-1β, and actin obtained pre-, at- and post-pressure.

**Figure 4:**
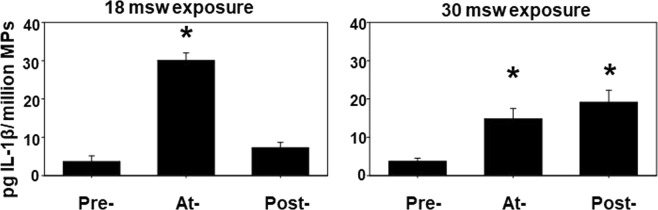
IL-1β in MPs. Data show the concentration of IL-1β as pg/1 million MPs from blood obtained as described in the caption for Fig. 1. Results in Fig. 3 and prior work have shown that virtually all blood-borne IL-1β is found within MPs^11^. Data are mean ± SE, n is shown for each sample, * indicates significantly different from pre-exposure, p < 0.05, ANOVA.

Plasma samples centrifuged to pellet MPs and subjected to Western blotting demonstrated that virtually all IL-1β was present in the MPs pellet (Fig. 3), just as reported in the murine model^11^. Changes in the concentration of IL-1β in MPs related to diving are shown in Fig. 4. Content of IL-1β was significantly elevated during the high air pressure exposures at both 18 and 30 msw, and following decompression from 30 msw.

### *Ex vivo* studies of neutrophils and platelets

**Figure 5:**
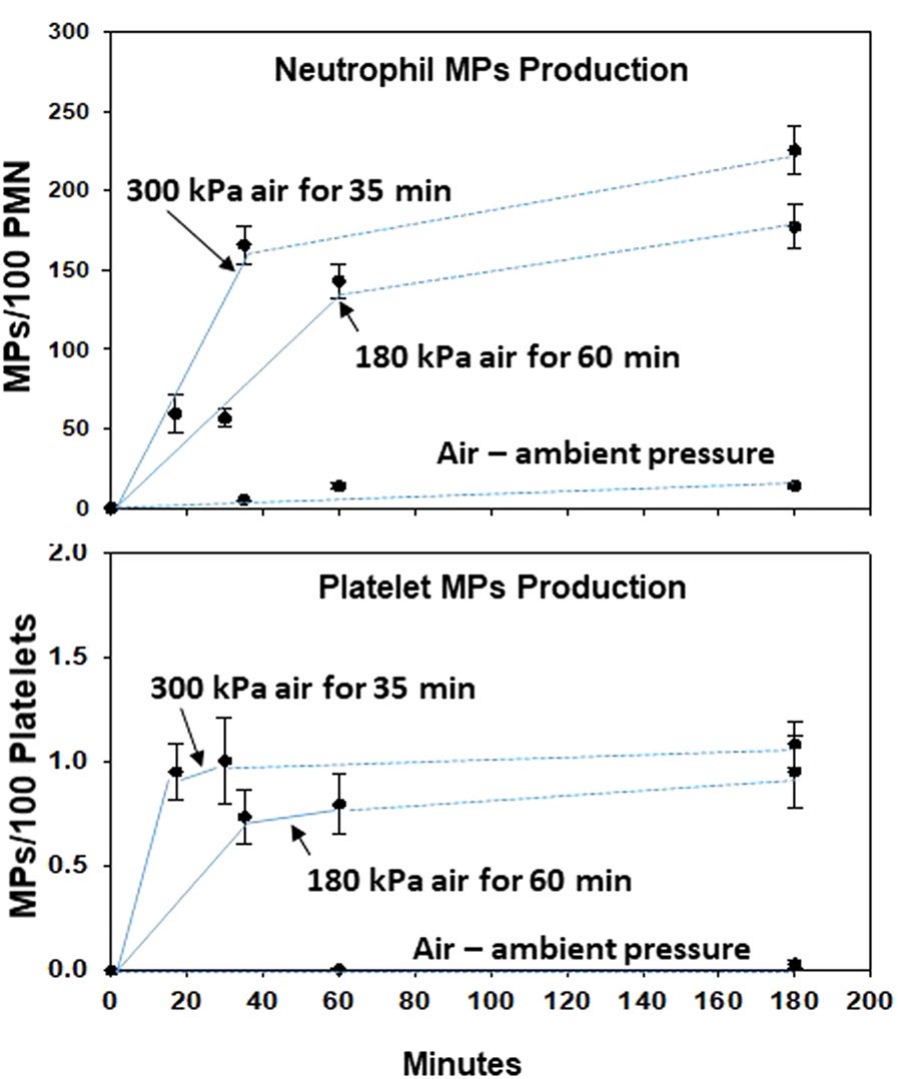
MPs generation by neutrophils and platelets incubated *ex vivo*. Samples prepared from research subjects were incubated following the same pressure profiles as used in the simulated dive subjects, 180 kPa air for 1 hour or 300 kPa for 35 minutes, then decompressed and left at ambient pressure for 2 hours. At intervals the number of MPs was measured and plotted as number per 100 neutrophils (PMN) or platelets in the suspensions. Solid lines in the figure indicate samples that were kept at pressure for the indicated time and fixed immediately on decompression, dotted lines indicate samples incubated in air at ambient pressure. Data are mean ± SE, * indicates significantly different from pre-exposure, p < 0.05, ANOVA.

**Figure 6:**
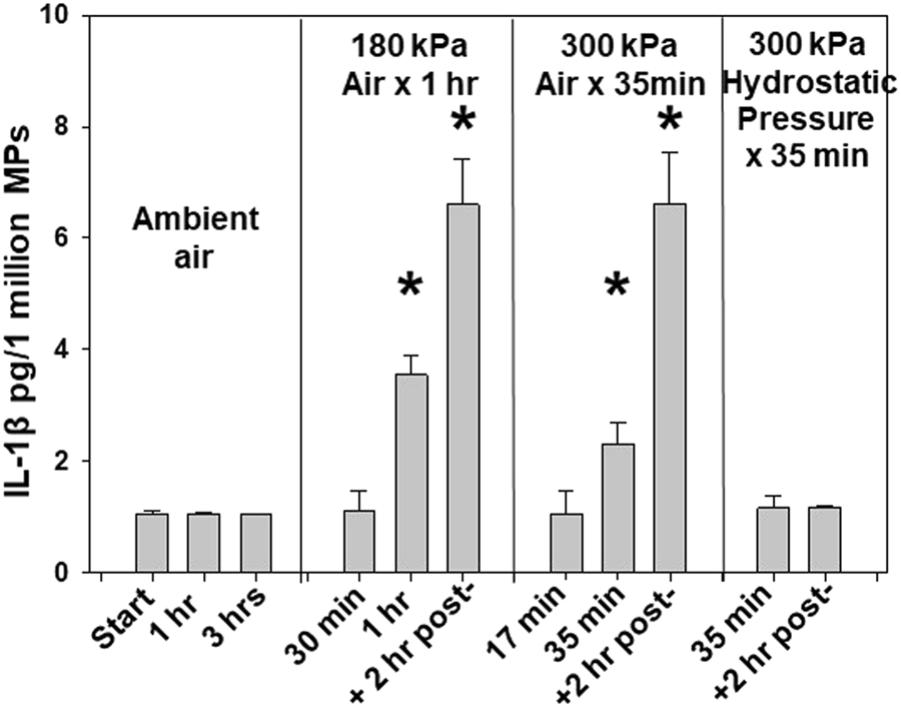
IL-1β in neutrophil MPs generated *ex vivo*. Data show the concentration of IL-1β as pg/1 million MPs in samples obtained as described in the caption for Fig. 5. Data are mean ± SE, * indicates significantly different from pre-exposure (labeled as Start), p < 0.05, ANOVA.

We next evaluated whether the gas pressure profiles used with divers stimulated production of MPs by isolated neutrophils and platelets. Samples from 8 research subjects were suspended in buffer and exposed to air pressure in the same manner described above, or simply to hydrostatic pressure with no gas phase. Results (Fig. 5) demonstrate generation of MPs during exposure to gas pressure, with nominal further increase after decompression. There were no increases in MPs by samples exposed to hydrostatic pressure at 300 mPa. ELISA assays (Fig. 6) demonstrated elevations of neutrophil intra-particle IL-1β concentrations. Notably, the pattern of elevations differed from the increase in particle number, as a marked increase in concentrations occurred during the 2 hours incubation post-decompression. No significant elevations in rate of MPs generation of IL-1β concentration occurred with exposures to hydrostatic pressure (no gas phase). IL-1β was not detected in platelet-derived MPs under any conditions.

## Discussion

Results demonstrate that in humans, exposures to high gas pressures trigger production of MPs containing elevated concentrations of IL-1β and neutrophil activation. *Ex vivo* studies confirm that these gas pressure profiles stimulate neutrophils and platelets to produce MPs, and that neutrophil MPs contain elevated concentrations IL-1β. This is the first study with a possible pathophysiological relationship to DCS showing that human responses to diving occur *prior* to decompression.

Previous work with platelets and neutrophils *ex vivo* has shown that reactive oxygen species generated during exposures to high partial pressures of helium, nitrogen, argon or CO_2_ gas trigger a cascade of intracellular changes that generate MPs^16,26,27^. Studies with the murine model suggest that neutrophil activation occurs prior or concurrent with MPs production and NLRP3 inflammasome activation, although we cannot rule out the possibility that MPs and IL-1β may be a stimulus for neutrophil activation in human divers. In the 30 msw exposure, it is also possible that platelet interactions with the neutrophil membrane could cause activation.

The variability within our pre-dive data is comparable to that observed in human populations studied for reasons other than diving, and similar to results observed in divers without DCS^22,28–30^. However, it is greater than we have observed in several prior investigations^6,7,10,31,32^. A recent study reported that the blood transcriptome indicative of apoptotic, inflammatory, and innate immune responses in non-divers differed from experienced divers, even though they had abstained from diving for over 2 weeks^33^. It is possible that some of the differences between groups in our study was related to activities performed prior to our study, as the research subjects (all military divers) had abstained from diving activities for at least 1 week before the research exposures.

Many changes, especially in the 18 msw group, resolved within the 2 hours post-decompression period. Clearance of MPs seems a likely explanation as the *ex vivo* studies demonstrated no loss of MPs in the 2 hours post-decompression. Intracellular events may also limit MPs production. The cytoskeletal modifications required for coupled MPs and IL-1β production are transient and suppressed by anti-oxidant mechanisms^34,35^. We have seen more persistent changes after 18 msw exposures in open water divers, an activity that causes more stress than simulated diving in a hyperbaric chamber^6^. We interpret the persistent changes in the 30 msw group as reflecting a more provocative exposure, although neither the 18 nor 30 msw groups reported DCS symptoms.

We did not perform Doppler-based assessments of intravascular bubbles in our subjects. MPs may serve as a nidus for the vascular gas emboli that are routinely seen in divers^12–14^. Some have reported a close association between MPs and bubbles, while others have not^7–10,31^. Whether MPs are the nucleation site for bubbles, or these are parallel events, remains unclear. However, results from this study are important as they highlight the need to consider biochemical events occurring during high pressure exposures, in addition to the more conventional perspective of inert gas uptake and liberation from body tissues according to Henry’s Law^36–39^. Further investigation is required to elucidate the association between MPs and bubbles, as well as mechanisms by which MPs containing IL-1β may contribute to the pathology of DCS.

## Methods

### Subjects

Canadian Armed Forces (CAF) Clearance Divers were recruited from Fleet Dive Unit (Atlantic) (FDU[A]), Fleet Dive Unit (Pacific)(FDU[P]) and the Experimental Dive and Undersea Group at Canadian Forces Environmental Medicine Establishment (CFEME). All subjects were deemed medically fit to undergo hyperbaric exposure by an Advanced Diving Medical Officer or Consultant in Diving and Submarine Medicine. Written informed consent for study participation was obtained from all subjects. Subjects abstained from alcohol for 24 hours, strenuous exercise for 48 hours, and hypo- or hyperbaric exposures (diving, flying, hyperbaric chamber, hypobaric chamber) for at least 7 days preceding the experimental dives. All procedures were approved by the Defence Research and Development Canada and University of Maryland Human Ethics Committees, and all experiments were performed in accordance with relevant guidelines and regulations. *Ex vivo* studies on neutrophils and platelets were performed using blood samples obtained from 8 research subjects following the same restrictions as the divers.

### Simulated dives

The subjects were exposed to simulated dives (high-pressure exposures) following Canadian Forces Standard Air Decompression Tables (DCIEM). Exposures to 18 msw were for 60 minutes, 30 msw for 35 minutes followed by staged decompression. These dives fall within normal operational limits of CAF diving, and they were conducted within hyperbaric chambers at FDU(A), FDU(P) and CFEME. No exertion or specific tasks were performed by the subjects during the simulated dive. The dives commenced at the same time of day +/− one hour to minimize diurnal variation in MP production. The multi-place chambers were compressed with air only and without the use of breathing masks to remove impact of carbon dioxide retention on results. No adverse effects were reported among research subjects.

### Blood acquisition

Blood (~5 mL) was drawn into Cyto-Chex BCT test tubes that contain a proprietary preservative (Streck Inc., Omaha, NE). Blood samples were obtained 30 minutes prior to pressurization, 10 minutes prior to the end of pressure exposure (“bottom time”) and 2 hours post-decompression. The samples were sent by express mail to the University of Maryland for analysis where processing occurred within 12 hours arrival and no more than approximately 72 hours after collection. Prior work has shown that diving-induced MPs and neutrophil characteristics remain unchanged when samples in Cyto-Chex BCT test tubes are processed within 3 weeks from time of acquisition^6^.

### Reagents

Chemicals were purchased from Sigma-Aldrich (St. Louis, MO) unless otherwise noted. Annexin binding buffer and the following agents were purchased from BD Pharmingen (San Jose, CA): fluorescein isothiocyanate (FITC) conjugated Annexin V, FITC-conjugated anti-human myeloperoxidase (MPO), BV421-conjugated anti-human CD66b, P- phycoerythrin (PE) conjugated anti-human CD31, R-PE conjugated anti-human CD18, and PerCP/Cy5.5 conjugated anti-human CD41.

### Procedures for *ex vivo* experiments

Neutrophils and platelets were isolated by centrifugation, suspended in buffer and exposed to pressure following the same procedures published in prior reports^16,26,27^. Hydrostatic pressure control studies were performed by placing cell suspensions in a plastic syringe so that pressure was transmitted to the suspensions via the moveable plunger without exposure to gas.

### Standard procedures for MPs isolation and analysis

All reagents and solutions used for MPs isolation and analysis were filtered with 0.1 µm filter (EMD Millipore, Billerica, MA). MPs were isolated and prepared for analysis as previously described^12,14^. Briefly, blood and suspensions from *ex vivo* studies were centrifuged for 5 min at 1,500 g. EDTA was added to the supernatant to achieve 12.5 mM to prevent MPs aggregation, centrifuged at 15,000 g for 30 min, and the supernatant analyzed by flow cytometry as described in previous publications^12,14^.

Flow cytometry was performed with an 8-color, triple laser MACSQuant® Analyzer (Miltenyi Biotec Corp., Auburn, CA) using MACSQuantify™ software version 2.5 to analyze data. MACSQuant was calibrated every other day with calibration beads ((Miltenyi Biotec Corp., Auburn, CA). Forward and side scatter were set at logarithmic gain. Photomultiplier tube voltage and triggers were optimized to detect sub-micron particles. Micro-beads of 3 different diameters 0.3 µm (Sigma, Inc., St. Louis, MO), 1.0 µm and 3.0 µm (Spherotech, Inc., Lake Forest, IL) were used for initial settings and before each experiment as an internal control. Samples were suspended in Annexin binding buffer solution (1:10 v/v in distilled water, (BD Pharmingen, San Jose, CA), and combined with FITC-conjugated annexin V and optimized concentrations of antibodies listed above. Analysis was carried out after establishing negative controls by the fluorescence-minus-one control test. All reagents and solutions used for MPs analysis were sterile and filtered (0.1 *μ*m filter). MPs were defined as annexin V-positive particles with diameters of 0.3 to 1 µm diameter (smaller particles were excluded as they include protein aggregates and cannot be reliably considered as vesicles). The concentration of MPs in sample tubes was determined by MACSQuant® Analyzer according to exact volume of solution from which MPs were analyzed.

### Neutrophil activation analysis

Whole fixed blood from the Cyto-chex tubes (100 µl) was stained for 30 min at room temperature in the dark with optimized concentrations of antibodies as listed above. After staining 2 ml phosphate buffered saline (PBS) was added to dilute each sample tube prior to analysis, with the cytometer acquisition set to use anti-human CD66b as the fluorescence trigger to recognize neutrophils.

### IL-1β measurements and related studies

A human-specific ELISA Kit (eBioscience, San Diego, CA) was used to evaluate the concentration of IL-1β in MPs samples following the manufacturer’s instructions. MPs were first prepared as described for flow cytometry but then centrifuged at 100,000 g for 60 min, and the MPs pellet lysed with 0.3 ml lysis buffer (20 mM Tris, 150 mM NaCl, 1% Nonidet P-40, 0.5% sodium deoxycholate, 1 mM EDTA and 0.1% SDS (pH 7.5) with protease inhibitors cocktail (Sigma)). The protein content of the sample was measured, diluted to 5 mg/ml, and 20 µg protein used for analysis. Some MPs samples were also subjected to Western blotting. Following the standard preparation of 15,000-*g* plasma supernatants, MPs were counted and a volume containing exactly 10,000 MPs was centrifuged at 21,000-*g* to sediment the MPs. The MPs pellets and supernatant from the same samples were then blotted to discern if IL-1β was within or outside MPs.

### Statistical analysis

Results are expressed as the mean ± SE and data compare by repeated measures analysis of variance (ANOVA) on ranks using SigmaStat (Jandel Scientific, San Jose, CA). Multiple comparisons were performed using Dunnett’s method and the level of statistical significance was defined as p < 0.05.

## Data Availability

The datasets generated during and/or analysed during the current study are available from the corresponding author on reasonable request.
